# Nordic Walking Training Causes a Decrease in Blood Cholesterol in Elderly Women Supplemented with Vitamin D

**DOI:** 10.3389/fendo.2018.00042

**Published:** 2018-02-20

**Authors:** Krzysztof Prusik, Jakub Kortas, Katarzyna Prusik, Jan Mieszkowski, Joanna Jaworska, Wojciech Skrobot, Marcin Lipinski, Ewa Ziemann, Jedrzej Antosiewicz

**Affiliations:** ^1^Gdańsk University of Physical Education and Sport, Faculty of Tourism and Recreation, Department of Health Promotion, Gdańsk, Poland; ^2^Gdańsk University of Physical Education and Sport, Faculty of Physical Education, Department of Biochemistry, Gdańsk, Poland; ^3^Gdańsk University of Physical Education and Sport, Faculty of Physical Education, Department of Physiology, Gdańsk, Poland; ^4^Gdańsk University of Physical Education and Sport, Faculty of Rabilitation and Kinesiology, Department of Kinesiology, Gdańsk, Poland; ^5^Department of Pharmaceutical Biochemistry, Medical University of Gdańsk, Gdańsk, Poland; ^6^Gdańsk University of Physical Education and Sport, Faculty of Rabilitation and Kinesiology, Department of Physiology and Pharmacology, Gdańsk, Poland; ^7^Department of Bioenergetics and Physiology of Exercise, Medical University of Gdańsk, Gdańsk, Poland

**Keywords:** physical fitness, exercise, LDL, HDL, health training

## Abstract

**Objective:**

Different studies have demonstrated that regular exercise can induce changes in the lipid profile, but results remain inconclusive. Available data suggest that correction of vitamin D deficiency can improve the lipid profile. In this study, we have hypothesized that Nordic Walking training will improve lipid profile in elderly women supplemented with vitamin D.

**Methods:**

A total of 109 elderly women (68 ± 5.12 years old) took part in the study. First group [experimental group (EG): 35 women] underwent 12 weeks of Nordic Walking (NW) training combined with vitamin D supplementation (4,000 IU/day), second group [supplementation group (SG): 48 women] was only supplemented with vitamin D (4,000 IU/day), and third group [control group (CG): 31 women] was not subject to any interventions. Blood analysis of total cholesterol (TC), triglycerides (TG), high-density lipoprotein cholesterol (HDL-C), low-density lipoprotein cholesterol (LDL-C), and 25-OH-D_3_ was performed at baseline and after the 12 weeks of NW training. Additionally, a battery of field tests specifically developed for older adults was used to assess the components of functional fitness. The same blood analysis was repeated for the EG 6 months after the main experiment.

**Results:**

After 12 weeks of NW training and vitamin D supplementation, in the EG a decrease in TC, LDL-C, and TG was observed. In the SG, no changes in the lipid profile were observed, whereas in the CG an increase in the HDL-C level was noticed. Positive physical fitness changes were only observed in the EG.

**Conclusion:**

Our obtained data confirmed baseline assumption that regular exercise induces positive alternations in lipid profile in elderly women supported by supplementation of vitamin D.

## Introduction

Regular exercise has been demonstrated to induce several adaptive changes manifested by an increase in the endurance strength of skeletal muscle. Positive changes in brain structure and function have also been observed in response to physical activity (1). These and other adaptive changes induced by exercise are known to lower the risk of cardiovascular disease, diabetes, cancer, depression, and many others (2), often associated with aging. The pro-healthy effect of physical activity on the risk of these diseases may be partially attributed to beneficial changes in insulin sensitivity, inflammatory markers, and blood lipids post (3, 4). However, the topic continues to raise questions; despite the fact that many studies have reported a positive effect of regular exercise on blood lipids, several studies have shown no effect at all (5).

Vitamin D is an endogenous hormone known to regulate expression of hundreds of genes. Since it is synthetized from 7-dehydrocholesyterol, it is also possible that its status is interrelated with cholesterol.

There is some evidence that apolipoprotein A-I (apo A-I) gene expression can be modified by vitamin D. At the same time, apo A-I is an essential component of high-density lipoprotein (HDL) molecules, positively influencing its quality. The effects of exercise on blood lipids have been shown to depend on applied dietary solutions or some drug compounds (6). In particular, vitamin D combined with exercise has been demonstrated to modify lipid metabolism and blood lipid profile by improving insulin sensitivity (7).

Physical activity itself is associated with better vitamin D status (25-OH-D_3_) (8); however, the effect of vitamin D status on blood lipids alone remains unclear. Supplementation of vitamin D at 400 IU for 5 years has been reported to induce no significant changes in blood lipids (9). Conversely, another study has shown that the concentration of 25-OH-D_3_ correlated inversely with triglycerides (TG) and total cholesterol (TC) (10). Based on collected data, we have hypothesized that beneficial effects of exercise on the lipid profile may be influenced by the vitamin D status.

Vitamin D deficiency or insufficiency is prevalent in most countries; it is now considered a pandemic (11, 12). Consequently, in this paper, we have studied effects of vitamin D supplementation alone and combined with 12 weeks of Nordic Walking (NW) training on the lipid profile in elderly women. The present study is the first published report to implicate effects of exercise and vitamin D on lipid profile in elderly subjects.

## Materials and Methods

Three groups of elderly women participated in the study, all aged over 60 years (68.4 ± 5.0 years old). They were randomly assigned to three groups. First group [experimental group (EG)] involved 35 women subjected to 12 weeks of NW training supported with vitamin D supplementation (average 4,000 IU/day). Second group [supplementation group (SG)] involved 48 women, subject only to vitamin D supplementation (average 4,000 IU/day). Vitamin D was supplemented three times per week with appropriate doses to reach 28,000 IU/week which is essentially concordant with current recommendations (13).

Third group [control group (CG)] involved 31 women, who did not receive any supplementation and did not participate in the training. Considering the pleiotropic function of vitamin D on many aspects of human health, we recognized that it would be unethical to include a placebo group in the experiment.

All subjects underwent a medical check-up prior to the experiment. Exclusion criteria from the study included the following: uncontrolled hypertension (systolic blood pressure over 140 mmHg and diastolic over 100 mmHg) a history of cardiac arrhythmia, cardio-respiratory disorders, and orthopedic problems. It was recommended that the volunteers did not change their lifestyle and diet habits throughout the study. Experiment activities were completed at the Gdansk University of Physical Education and Sport.

### Ethics Statement

The examination was officially approved by the Bioethical Committee of the Regional Medical Society in Gdansk (KB-26/14) according to the Declaration of Helsinki and was registered as a Clinical trail NCT03417700. Before commencing the training and testing, subjects received verbal description of the experiment. Written informed consent was signed by all participants. The ethics approval was also obtained for referring participants to their family physician upon detection of any abnormal pathology results during the medical check-up.

### Blood Analysis

Blood collection was performed following the same timeline for all groups: at baseline and one day directly after the 12-week training program. Additionally, in EG blood collection took place also 6 months after end of NW training program. Blood samples were obtained between 7 and 8 a.m. after an overnight fast. The serum was separated by centrifugation at 1,000× *g* for 15 min and stored at −80°C pending analysis. Red blood cells count (10^6^/μL) (RBC), hematocrit (%) (Hct), and blood hemoglobin concentration (g/dL) (Hb), low-density lipoprotein cholesterol level (LDL-C), HDL-C, TC, and triglyceride (TG) were determined from venous blood samples by conventional methods using a BIOSYSTEMS S.A, ANALYZER A25 Costa Brava, Barcelona, Spain.

### Vitamin D Assessment

Vitamin D metabolite 25-OH-D_3_ was measured by high-performance liquid chromatography mass spectrometry (HPLC-MS). The HPLC system was a Transcend TLX turboflow 2 system attached to a TSQ Quantum Ultra triple quadrupole mass spectrometer (Thermo Fisher Scientific, San Jose, CA, USA) as described before (14).

### Measurements of Physical Fitness

A battery of field tests developed specifically for older adults was used to assess components of functional fitness in the EG. These tests require very little time or equipment and are designed to be conducted in community settings. In accordance with Rikli and Jones (15, 16), we used the following tests at the beginning of the study and after the 12 weeks of training. The Senior Fitness Test (SFT) consists of the following six items: (1) 30-s chair stand, (2) arm curl, (3) chair sit-and-reach, (4) back scratch, (5) 2-min step, and (6) 8-foot up-and-go. The tests were performed in this order with 1 min of rest in-between. Before each test, the exercises were demonstrated for the participants, who made a trial attempt before completing the actual drill. The walking test was performed only once.

### Exercise Protocol

The same group of research assistants and instructors supervised all training sessions. The EG completed 12 weeks of a mesocycle exercise, divided into three microcycles. The training procedure was described in detail in our previous study (17). The participants met three times a week, 1 h after eating a light breakfast and performed the main session of NW training at 60–70% intensity of the maximal HR (10-min warm-up, 45–55-min NW, and 10-min cooldown). Each training unit was recorded with Garmin Forerunner 405 with a built-in GPS.

### Statistical Analysis

Statistical analysis was performed using Statistica 12.0 software (Statsoft, Tulsa, OK, USA). All values are expressed as mean ± SD. The Shapiro–Wilk test was applied to assess the homogeneity of dispersion from the normal distribution. The Brown–Forsythe test was used to evaluate the homogeneity of variance. For homogenous results, a paired *t*-test analysis was performed to identify significantly different results. For heterogeneous results, the Wilcoxon signed-rank test was applied. For homogenous results, the analysis of variance (ANOVA) for repeated measurements and the *post hoc* Tukey test for unequal sample sizes were performed to identify significantly different results. For heterogeneous results, the ANOVA Friedman’s test and right *post hoc* test was applied. The significance level was set at *p* < 0.05. The relations between variables were evaluated using the Pearson correlation coefficient.

## Results

### General Outcomes

Baseline descriptive characteristics of participants are summarized in Table 1. Values of the body mass index (BMI), percentage, and absolute fat tissue indicate that our groups were within the range of normal to slightly overweight. There were no significant changes in the body composition after the 12 weeks of NW training in the EG nor in the SG or CG.

**Table 1:** 12 weeks of Nordic Walking training had no effect on the body composition but had improved physical fitness in elderly women supplemented with vitamin D supplementation.

	Experimental group (exercise and supplement)			Control group		Supplemented group	
	Baseline (*n* = 35)	After 12 weeks (*n* = 35)	After 6 month (*n* = 21)	Baseline (*n* = 31)	After 12 weeks (*n* = 31)	Baseline (*n* = 48)	After 12 weeks (*n* = 48)
**Weight**(kg)	69.2 ± 10.7	69.0 ± 11.1	66.5 ± 7.9	70.7 ± 11.4	71.2 ± 11.8	69.2 ± 10.1	70.3 ± 10.0
**BMI**(kg•m^−2^)	26.0 ± 4.0	26.3 ± 4.0	25.6 ± 3.3	27.1 ± 3.7	27.5 ± 4.0	26.4 ± 3.5	26.8 ± 3.7
**Fat**(kg)	24.8 ± 8.1	24.9 ± 8.6	23.3 ± 6.4	27.0 ± 7.4	27.1 ± 8.0	25.1 ± 7.3	25.9 ± 7.7
**Fat**(%)	35.3 ± 7.1	35.2 ± 7.7	34.5 ± 6.2	37.3 ± 6.0	37.0 ± 5.7	35.5 ± 6.7	36.1 ± 7.1
**FFM**(kg)	44.2 ± 5.1	44.1 ± 4.9	43.2 ± 3.6	43.7 ± 5.6	44.1 ± 5.6	44.2 ± 5.0	44.6 ± 5.1
**TBW**(kg)	32.5 ± 3.8	32.4 ± 3.6	31.7 ± 2.6	32.1 ± 4.1	32.4 ± 4.1	32.5 ± 3.7	32.7 ± 3.8
**Chair stand**(no. of stands)	21 ± 4	22 ± 3	22 ± 3	19 ± 5	20 ± 5	16 ± 3	17 ± 4
**Arm curl**(no. of reps)	26 ± 5	28 ± 6^a^	29 ± 3	26 ± 7	26 ± 6	24 ± 5	23 ± 5
**Chair sit-&-reach** (cm)	7 ± 9	9 ± 9	6 ± 9	4 ± 10	4 ± 10	6 ± 10	4 ± 10
**Back scratch** (cm)	−1 ± 8	0 ± 8	1 ± 7	−1 ± 7	−1 ± 8	−1 ± 7	−2 ± 6
**8-foot up-&-go** (s)	4.72 ± 0.5	4.24 ± 0.4^a^	4.56 ± 0.7	4.66 ± 0.7	4.61 ± 0.7	4.49 ± 0.9	4.61 ± 0.7

*Values are means (±SD). BMI, body mass index; Fat, fat mass; Fat%, percentage of body fat; FFM, free fat mass; TBW, total body water*.

*^a^Significant differences from baseline*.

### Level of Physical Fitness

The 12 weeks of NW training improved all measured fitness parameters. Specifically, changes in the level of general shoulder coordination and flexibility were statistically significant (Table 1). The applied training program also improved the level of endurance and lowered the heart rate at baseline [from 81 ± 14 to 77 ± 15 bpm, average values during exercise (120 ± 17 to 116 ± 16 bmp) and after the exercise (141 ± 22 to 133 ± 23 bmp)]. These changes, however, were not statistically significant. The level of physical fitness in the SG and CG was lower as compared with the EG; however, the differences did not reach statistical significance (Table 1). In addition, some improvement in endurance has been observed in 2,000-m test (1,082 ± 108 vs. 1,059 ± 116 s); however, it did not reach statistical significance (*p* = 0.2, CI − 49 to 11).

### General Characteristics of Blood Tests

Hematological parameters of the EG were within reference ranges in all subjects at baseline as well as after the training. Nonetheless, a significant drop in Hb, mean corpuscular hemoglobin concentration (MCHC), and mean corpuscular hemoglobin (MCH) was observed after training (Table 2). Importantly, no iron deficiency (not shown) or anemia was observed in any subject.

**Table 2:** Hematological parameters in the experimental group.

Variable	Baseline	After 12 weeks	*p*-Value	CI– 95%	CI+ 95%
**Hb** (g·dL^−1^)	14.0 ± 0.9	13.7 ± 1.0^a^	0.03	−0.55	−0.02
**MCH** (pg)	30.1 ± 1.0	29.8 ± 1.1^a^	0.00	−0.52	−0.13
**MCHC** (g·dL^−1^)	34.0 ± 0.8	33.5 ± 0.8^a^	0.00	−0.64	−0.26
**HCT** (%)	41.1 ± 2.7	40.8 ± 2.6	0.42	−1.09	0.47
**MCV** (fL)	88.6 ± 3.1	88.9 ± 3.4	0.14	−0.07	0.56
**RBC** (min·μL^−1^)	4.6 ± 0.3	4.6 ± 0.3	0.28	−0.14	0.04

*Values are means (±SD). CI, confidence interval; Hb, hemoglobin; MCH, mean cell hemoglobin; MCHC, mean corpuscular hemoglobin concentration; HCT, hematocrit; MCV, mean corpuscular volume; RBC, erythrocytes. After 12-weeks of comparison between baseline and after the whole period of Nordic Walking training*.

*^a^Significantly different from baseline*.

### Lipid Profile

Vitamin D supplementation combined with the 12 weeks of NW training induced significant changes in the lipid profile in the EG. A significant decrease in TC, LDL-C, and TG was noted. All these changes were accompanied by a significant rise in 25-OH-D_3_ concentration owing to the applied supplementation. The training and supplementation caused a decrease in HDL-C; however, the shift was not statistically significant (Table 3). A detailed analysis of the ratios between parameters of the lipid profile (LDL-C/HDL-C, TC/HDL-C, TC/LDL-C, TG/HDL-C) showed no additional tendency for change, neither within individual groups nor in the intergroup comparison. Interestingly, 6 months following the experiment, the levels of TC, HDL-C, and LDL-C returned to baseline in the EG (Table 3).

**Table 3:** Changes in lipid profile in elderly women when Nordic Walking training is combined with vitamin D supplementation.

	Baseline (*n* = 35)	After 12 weeks of NW training and vit D supplementation (*n* = 35)	*p*	CI (−95%; +95%)	After 6 month without training and vit D supplementation (*n* = 21)
**TC** (mg/dL)	228.8 ± 36.0	207.7 ± 37.4^a^	0.00	(−34.2; −7.9)	239.0 ± 50.3
**HDL-C** (mg/dL)	70.8 ± 19.3	67.6 ± 18.5	0.12	(−7.4; 0.9)	73.2 ± 20.5
**LDL-C** (mg/dL)	134.5 ± 29.6	121.1 ± 32.2^a^	0.02	(−24.5; −2.3)	143.7 ± 47.4
**TG** (mg/dL)	117.1 ± 55.6	94.6 ± 34.0^a^	0.00	(−36.4; −8.5)	109.8 ± 46.8
**LDL-C/HDL-C**	2.1 ± 0.6	1.9 ± 0.7	0.49	(−0.2; 0)	2.1 ± 0.8
**TC/HDL-C**	3.4 ± 0.8	3.2 ± 0.7	0.10	(−0.4; 0)	3.4 ± 0.9
**TC/LDL-C**	1.7 ± 0.2	1.8 ± 0.2	0.53	(0; 0.2)	1.7 ± 0.3
**TG/HDL-C**	1.9 ± 1.2	1.6 ± 0.8	0.09	(0; 0.6)	1.7 ± 1.0
**Vit D** (ng/mL)	20.8 ± 7.7	38.4 ± 14.3^a^	0.00	(12.6; 22.6)	23.6 ± 10.8

*Values are means (±SD). TC, total cholesterol; HDL-C, high-density lipoprotein; LDL-C, low-density lipoprotein; TG, triglycerides*.

*^a^Significant differences from baseline*.

In the CG, the concentration of 25-OH-D_3_ remained stable over the 12-week period, considerably lower compared with the EG and SG. An increase in HDL concentration was the only change observed in the CG (Table 4). At the same time, in the SG, an increase in 25-OH-D_3_ was accompanied by a decrease in HDL-C relative to the CG (Table 4).

**Table 4:** Effect of vitamin D supplementation on lipid profile in elderly women.

	Control group		Supplemented group	
	Baseline (*n* = 31)	After 12 weeks (*n* = 31)	Baseline (*n* = 48)	After 12 weeks (*n* = 48)
**TC** (mg/dL)	215.5 ± 41.6	235.0 ± 52.2	218.2 ± 57.3	212.2 ± 56.4
**HDL-C** (mg/dL)	73.2 ± 16.1	79.8 ± 16.3^a^^,^^b^	70.1 ± 14.6	69.1 ± 13.3**^b^**
**LDL-C** (mg/dL)	121.5 ± 38.4	133.9 ± 51.6	127.0 ± 54.9	121.0 ± 53.7
**TG** (mg/dL)	103.7 ± 32.4	106.7 ± 31.7	107.0 ± 37.4	110.5 ± 44.8
**LDL-C/HDL-C**	1.8 ± 0.7	1.8 ± 0.8	1.9 ± 1.2	1.8 ± 1.2
**TC/HDL-C**	3.1 ± 0.8	3.0 ± 0.9	3.2 ± 1.2	3.2 ± 1.3
**TC/LDL-C**	1.8 ± 0.3	1.9 ± 0.4	1.9 ± 0.4	1.9 ± 0.4
**TG/HDL-C**	1.5 ± 0.7	1.4 ± 0.6	1.5 ± 0.6	1.6 ± 0.8
**Vit D** (ng/mL)	24.2 ± 12.1	24.6 ± 10.9	18.1 ± 9.1	40.7 ± 12.1^a^^,^^b^

*Values are means (±SD). TC, total cholesterol; HDL-C, high-density lipoprotein; LDL-C, low-density lipoprotein; TG, triglycerides*.

*^a^Significant differences from baseline*.

*^b^Significant differences between groups*.

## Discussion

Data obtained through this study suggest that regular exercise induced positive changes in the lipid profile in elderly women. We demonstrate that NW training combined with vitamin D supplementation led to a significant decrease in total blood cholesterol in elderly women. As reviewed by Leon and Schantz, many studies have demonstrated that exercise applied alone induced a decrease in LDL and TG, but had no effect on blood TC (18). Conversely, a 12-week program of aerobic exercise did not influence blood lipids despite triggering a decrease in TG and a transient increase in HDL (19). It has also been revealed that the lipid-profile concentrations: serum TG, TC HDL-C, and LDL-C did not differ between athletes and non-athletes (20). In our previous study, we have shown that 4 weeks of regular training in young rowers did not influence the lipid profile unless the subjects were supplemented with vitamin D (21). All of these data indicate that effects of exercise on blood lipids can be modulated by other factors, among many vitamin D. Our preliminary data on 25-OH-D_3_ demonstrate that most of the women participating in the study had exhibited vitamin D deficiency or insufficiency. Thus, in our present study, we have investigated the effect of regular training in combination with vitamin D supplementation.

The present study also demonstrates that NW training combined with vitamin D supplementation induced not only changes in total blood cholesterol, but also a significant decrease in LDL-C, yet no shift in HDL-C. Still, a comparison between the SG and the CG shows that vitamin D has a tendency to lower HDL-C. A previously published study supports this observation, as a low dose of vitamin D supplementation (300 IU/day) was shown to lead to a significant drop in HDL-C in postmenopausal women (22). In addition, no differences in the lipid profile between physically active (>3 h exercise/week) and physically inactive (<3 h exercise/week) women were observed. The authors concluded that vitamin D supplementation may have an unfavorable effect on lipids in postmenopausal women undergoing hormone replacement therapy (22). It is difficult to agree with this conclusion as a study published in recent years demonstrated that it is the quality rather than quantity of HDL that plays an important role in human health (23–25). The level of HDL in people converted to low-fat high-carbohydrate diet was observed to decrease, while the atheroprotective potential improved (26). Another study has shown the level of HDL-C to decline in bariatric patients after surgical intervention, reaching the preoperative level after 6 months. It has been demonstrated that that during this period a qualitative switch took place as apoE HDL was replaced by apoA-I HDL (25). The effect of vitamin D on the apoA-I gene expression is debatable because both positive and negative shifts have been reported in response to 1,25-OH-D_3_ treatment. These data indicate that the effects of vitamin D on blood lipids can be modulated by other factors.

At the same time, in our previously published study we have demonstrated that regular exercise had no effect on the HDL-C level in vitamin-D-deficient young men, but led to its significant reduction when accompanied by vitamin D supplementation (21). It is generally believed that changes are blood lipids result from the adaptive response of skeletal muscle, manifested in an increase in lipid oxidation, and insulin sensitivity (27). We observed some improvement in endurance in the EG, which could have been accompanied by an increase in the mitochondrial oxidative potential. Contrary to our expectation, changes in endurance capacity were accompanied with significant decrease in Hb and MCH; however, all the recorded data were in reference range. More research is needed to understand the nature of these changes. Our data suggest that the shifts in blood lipids induced by NW training were mediated by vitamin D and some factors possibly discharged from exercising muscles (4). Interestingly, in the EG, in the 6-month period after the intervention, during which neither regular training nor supplementation were taking place, all lipids parameters and 25-OH-D_3_ returned to baseline values. This happened despite the fact that all participants had attended a talk about benefits of vitamin D and had received recommendations about vitamin D supplementation and exercise. Certainly, however, it is too early to judge the nature of these changes without data about subpopulation of HDL particles. The NW training and vitamin D supplementation had also a positive effect on blood TG. These data are in agreement with a previously published study, where plasma 25-OH-D was inversely associated with TG and TC (10, 28). Certainly, the main limitation of this study is the lack of a placebo group. As mentioned above, we decided not to include a placebo group in our study because of two main reasons. Firstly, most women taking part in our study were vitamin D deficient at the beginning of the experiment. Given that vitamin D deficiency can increase the risk of many morbidities, we decided that maintaining this state would be unethical. Secondly, observations made in this study are supported by our earlier research on young athletes (21). We can, thus, conclude that regular NW exercise induced positive changes in blood lipids in elderly women, in whom vitamin D deficiency was corrected.

## Ethics Statement

The examination was officially approved by the Bioethical Committee of the Regional Medical Society in Gdansk (KB-26/14) according to the Declaration of Helsinki.

## Author Contributions

KrP and JA designed the study and performed the research. JK, JA and EZ performed the research and wrote the paper. KaP, JM, JJ, WS, and ML performed the research.

## Conflict of Interest Statement

The authors declare that the research was conducted in the absence of any commercial or financial relationships that could be construed as a potential conflict of interest.

## References

[1] Chaddock L Erickson KI Prakash RS Kim JS Voss MW Vanpatter M A neuroimaging investigation of the association between aerobic fitness, hippocampal volume, and memory performance in preadolescent children. Brain Res (2010) 1358:172–83. 10.1016/j.brainres.2010.08.049 20735996 PMC3953557

[2] Manson JE Greenland P LaCroix AZ Stefanick ML Mouton CP Oberman A Walking compared with vigorous exercise for the prevention of cardiovascular events in women. N Engl J Med (2002) 347:716–25. 10.1056/NEJMoa021067 12213942

[3] Marusiak J Zeligowska E Mencel J Kisiel-Sajewicz K Majerczak J Zoladz JA Interval training-induced alleviation of rigidity and hypertonia in patients with Parkinson’s disease is accompanied by increased basal serum brain-derived neurotrophic factor. J Rehabil Med (2015) 47:372–5. 10.2340/16501977-1931 25510618

[4] Zoladz JA Majerczak J Zeligowska E Mencel J Jaskolski A Jaskolska A Moderate-intensity interval training increases serum brain-derived neurotrophic factor level and decreases inflammation in Parkinson’s disease patients. J Physiol Pharmacol (2014) 65:441–8. 24930517

[5] Paoli A Pacelli QF Moro T Marcolin G Neri M Battaglia G Effects of high-intensity circuit training, low-intensity circuit training and endurance training on blood pressure and lipoproteins in middle-aged overweight men. Lipids Health Dis (2013) 12:131. 10.1186/1476-511X-12-131 24004639 PMC3846819

[6] Pietila M Malminiemi K Huupponen R Rouru J Pulkki K Pere E Celiprolol augments the effect of physical exercise on insulin sensitivity and serum lipid levels in chronic heart failure. Eur J Heart Fail (2000) 2:81–90. 10.1016/S1388-9842(00)00054-4 10742707

[7] Chiu KC Chu A Go VL Saad MF. Hypovitaminosis D is associated with insulin resistance and beta cell dysfunction. Am J Clin Nutr (2004) 79:820–5. 10.1093/ajcn/79.5.820 15113720

[8] Giovannucci E Liu Y Rimm EB Hollis BW Fuchs CS Stampfer MJ Prospective study of predictors of vitamin D status and cancer incidence and mortality in men. J Natl Cancer Inst (2006) 98:451–9. 10.1093/jnci/djj101 16595781

[9] Rajpathak SN Xue X Wassertheil-Smoller S Van Horn L Robinson JG Liu S Effect of 5 y of calcium plus vitamin D supplementation on change in circulating lipids: results from the Women’s Health Initiative. Am J Clin Nutr (2010) 91:894–9. 10.3945/ajcn.2009.28579 20181812 PMC2844677

[10] Garcia-Bailo B Da Costa LA Arora P Karmali M El-Sohemy A Badawi A. Plasma vitamin D and biomarkers of cardiometabolic disease risk in adult Canadians, 2007-2009. Prev Chronic Dis (2013) 10:E91. 10.5888/pcd10.120230 23742939 PMC3682811

[11] Pludowski P Ducki C Konstantynowicz J Jaworski M. Vitamin D status in Poland. Pol Arch Med Wewn (2016) 126:530–9. 10.20452/pamw.3479 27509842

[12] Pludowski P Grant WB Bhattoa HP Bayer M Povoroznyuk V Rudenka E Vitamin D status in central Europe. Int J Endocrinol (2014) 2014:589587. 10.1155/2014/589587 24790600 PMC3984788

[13] Pludowski P Holick MF Grant WB Konstantynowicz J Mascarenhas MR Haq A Vitamin D supplementation guidelines. J Steroid Biochem Mol Biol (2018) 175:125–35. 10.1016/j.jsbmb.2017.01.021 28216084

[14] Gmiat A Mieszkowski J Prusik K Prusik K Kortas J Kochanowicz A Changes in pro-inflammatory markers and leucine concentrations in response to Nordic Walking training combined with vitamin D supplementation in elderly women. Biogerontology (2017) 18(4):535–48. 10.1007/s10522-017-9694-8 28316011 PMC5514208

[15] Jones CJ Rikli RE Beam WC. A 30-s chair-stand test as a measure of lower body strength in community-residing older adults. Res Q Exerc Sport (1999) 70:113–9. 10.1080/02701367.1999.10608028 10380242

[16] Rikli RE Jones CJ. Development and validation of criterion-referenced clinically relevant fitness standards for maintaining physical independence in later years. Gerontologist (2013) 53:255–67. 10.1093/geront/gns071 22613940

[17] Kortas J Kuchta A Prusik K Prusik K Ziemann E Labudda S Nordic walking training attenuation of oxidative stress in association with a drop in body iron stores in elderly women. Biogerontology (2017) 18:517–24. 10.1007/s10522-017-9681-0 28229255 PMC5514214

[18] Leon AS Sanchez OA. Response of blood lipids to exercise training alone or combined with dietary intervention. Med Sci Sports Exerc (2001) 33:S502–15. 10.1097/00005768-200106001-00021 11427777

[19] Leon AS Casal D Jacobs D Jr. Effects of 2,000 kcal per week of walking and stair climbing on physical fitness and risk factors for coronary heart disease. J Cardiopulm Rehabil (1996) 16:183–92. 10.1097/00008483-199605000-00006 8761839

[20] Petridou A Lazaridou D Mougios V. Lipidemic profile of athletes and non-athletes with similar body fat. Int J Sport Nutr Exerc Metab (2005) 15:425–32. 10.1123/ijsnem.15.4.425 16286673

[21] Jastrzebski Z Kortas J Kaczor K Antosiewicz J. Vitamin D supplementation causes a decrease in blood cholesterol in professional rowers. J Nutr Sci Vitaminol (Tokyo) (2016) 62:88–92. 10.3177/jnsv.62.88 27264092

[22] Heikkinen AM Tuppurainen MT Niskanen L Komulainen M Penttila I Saarikoski S. Long-term vitamin D3 supplementation may have adverse effects on serum lipids during postmenopausal hormone replacement therapy. Eur J Endocrinol (1997) 137:495–502. 10.1530/eje.0.1370495 9405029

[23] Tsompanidi EM Brinkmeier MS Fotiadou EH Giakoumi SM Kypreos KE. HDL biogenesis and functions: role of HDL quality and quantity in atherosclerosis. Atherosclerosis (2010) 208:3–9. 10.1016/j.atherosclerosis.2009.05.034 19595353

[24] Karavia EA Zvintzou E Petropoulou PI Xepapadaki E Constantinou C Kypreos KE. HDL quality and functionality: what can proteins and genes predict? Expert Rev Cardiovasc Ther (2014) 12:521–32. 10.1586/14779072.2014.896741 24650316

[25] Zvintzou E Skroubis G Chroni A Petropoulou PI Gkolfinopoulou C Sakellaropoulos G Effects of bariatric surgery on HDL structure and functionality: results from a prospective trial. J Clin Lipidol (2014) 8:408–17. 10.1016/j.jacl.2014.05.001 25110222

[26] Roberts CK Ng C Hama S Eliseo AJ Barnard RJ. Effect of a short-term diet and exercise intervention on inflammatory/anti-inflammatory properties of HDL in overweight/obese men with cardiovascular risk factors. J Appl Physiol (2006) 101:1727–32. 10.1152/japplphysiol.00345.2006 16902063

[27] Pedersen BK Saltin B. Evidence for prescribing exercise as therapy in chronic disease. Scand J Med Sci Sports (2006) 16(Suppl 1):3–63. 10.1111/j.1600-0838.2006.00520.x 16451303

[28] Sun X Cao ZB Tanisawa K Ito T Oshima S Ishimi Y Associations between the serum 25(OH)D concentration and lipid profiles in Japanese men. J Atheroscler Thromb (2015) 22:355–62. 10.5551/jat.26070 25346256

